# Accelerating patient recruitment using social media: Early adopter experience from a good clinical practice-monitored randomized controlled phase I/IIa clinical trial on actinic keratosis

**DOI:** 10.1016/j.conctc.2023.101245

**Published:** 2023-12-20

**Authors:** Vinzent Kevin Ortner, John R. Zibert, Olena Budnik, Ionela Manole, Charlotte Amalie Pind Laugesen, Signe Havsager, Merete Haedersdal

**Affiliations:** a Department of Dermatology, Copenhagen University Hospital, Bispebjerg and Frederiksberg, DK-2400, Copenhagen, Denmark; b Studies&Me A/S, Copenhagen, Denmark; c Coegin Pharma AB, Lund, Sweden; d Colentina Clinical Hospital, 2nd Department of Dermatology, Bucharest, Romania

## Abstract

**Background:**

Patient recruitment is a major cause of delays in randomized controlled trials (RCT). Online recruitment is evolving into an alternative to conventional in-clinic recruitment for RCT. The objective of this study was to test the effectiveness of online patient recruitment for an RCT on actinic keratosis (AK).

**Methods:**

In this proof-of-concept study, adults with AK were recruited for a Phase I/IIa RCT (NCT05164393) via social media using targeted advertising Interested users were directed to a landing page to learn about the study, respond to questionnaires, and upload self-obtained smartphone pictures of potential AK. Facebook Analytics was used to track the number of advertisement views, individual users exposed to the advertisement, and advertisement clicks. Following eligibility-review by remote dermatologists, candidates were contacted for an in-clinic visit. A review of pertinent RCTs on AK (2012–2022) was conducted to compare recruitment metrics.

**Results:**

The online campaign served 886,670 views, reached 309,000 users, and generated 27,814 clicks. A total of 556 users underwent eligibility review, leading to 140 pre-evaluated potential study subjects. The RCT's enrollment target of 60 patients (68.8 ± 7.1 years, 43.3 % female) was reached in 53 days after screening 90 participants in-clinic, corresponding to a screen failure rate of 33.3 %. The total cost of this online recruitment campaign was 14,285 USD i.e. 238 USD per randomized patient. Compared to the existing literature (44 RCTs), our online approach resulted in 9 times more time-efficient recruitment per site.

**Conclusion:**

Using targeted advertisements, 60 patients with AK were recruited for a single-center Phase I/IIa RCT in 53 days. Social media appears to be an efficient platform for online recruitment of patients with low-grade AK for RCT.

## 1 Introduction

Currently, recruitment for clinical trials is the single biggest cause of trial delays, with around 70 % of trials failing to meet their initial enrollment target and timeline [1]. Consequently, clinical trials are either prolonged to meet their intended enrollment target resulting in increased costs, or terminated early, affecting data quality and ultimately slowing down the development of new treatments [2].

Historically, studies on actinic keratosis (AK), precancerous skin lesions, depended on physician referrals and in-site examination, but with the growing use of social media and digital solutions, online recruitment may present a more time-efficient and cost-effective approach. In recent years, there has been an interest in using alternative approaches such as social media in combination with traditional recruitment methods to attract study participants as part of decentralizing and digitizing study activities, and as a strategy to potentially improve the effectiveness and overall quality of clinical trials [2]. Despite a rapidly growing body of evidence on the use of online recruitment for observational studies, experience with the use of targeted social media advertising and remote eligibility assessment for interventional, GCP-monitored trials on AK is lacking.

This paper investigates the effectiveness of using a digital ecosystem to recruit patients with AK required for a single-center GCP-monitored Phase I/IIa hybrid decentralized clinical study. In contrast to conventional recruitment, the herein-described online recruitment flow is initiated by social media users, who volunteer to participate in a virtual eligibility check before screening and enrollment as study patients in-clinic.

## 2 Materials and methods

**Fig. 1:**
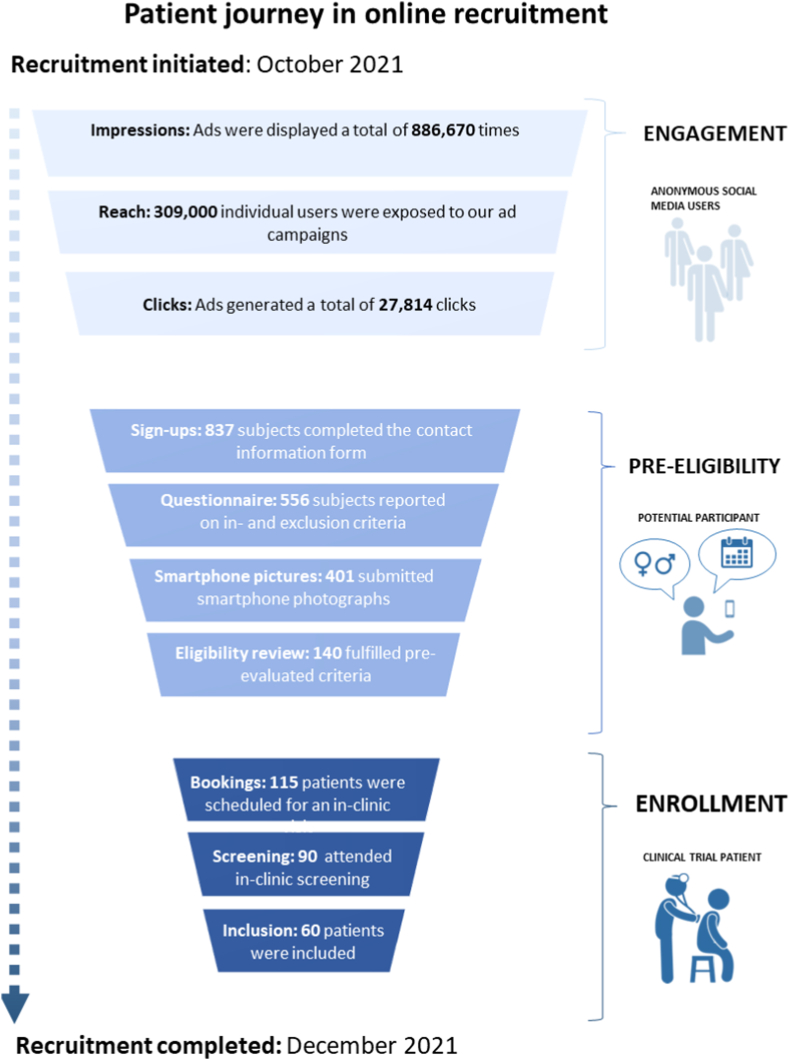
**Schematic overview of the patient journey in online recruitment in the Copenhagen Actinic Keratosis Study (COAKS).** Targeted social media advertising used to attract and engage users followed by a virtual pre-eligibility assessment can accelerate the enrolment of patients for early phase randomized controlled trials. In COAKS, 60 patients were enrolled in under 8 weeks.

*The data presented in this paper describes the recruitment for the COpenhagen Actinic Keratosis Study (COAKS), a 12-week single-center, GCP-monitored, randomized, vehicle-controlled, double-blind Phase I/IIa clinical hybrid trial in adults with multiple AK lesions Olsen grade 1 or 2,* with *the target* of *enrolling* *sixty participants. The trial was authorized by the Danish Medicines Agency (2021032485) and the Capital Region of Denmark's Ethics Committee (H-21018064), and registered on* *clinicaltrials.gov* *(NCT05164393). The trial was monitored in accordance with the International Council for Harmonization (ICH) Good Clinical Practice guideline and all applicable regulatory requirements.* Fig. 1 *shows a schematic recruitment flow;* supplementary material 1 *shows a CONSORT chart covering the enrollment phase, including the online eligibility check.*

### 2.1 Recruitment process

*Adults with self-reported AK living in a radius of* 75 km *-* 80 km *from the clinical site (Copenhagen University Hospital, Bispebjerg, Copenhagen, Denmark) were recruited* on *two social media platforms Facebook and Instagram (Meta, Dublin, Ireland), using targeted social media campaigns with different versions of paid online advertisements. An overview of all filters and settings, as well as in- and exclusion criteria, can be found in* supplementary material 2 *and our published study protocol.* [3] *Each advertisement was composed of an image, a title, and a brief message of fewer than 200 characters designed to generate traffic on the platforms.* A total of 20 different Facebook advertisements were created and combined in eight different campaigns, which were active for approximately one week at a time. Social media campaigns were run sequentially to avoid competing for the same target audience which might have increased recruitment costs. *By* *clicking the advertisement, possible candidates were directed to a study-specific landing page that contained high-level information about the study, and if interested, they followed a two-step process. Firstly, they were asked to sign up for the recruitment platform providing their contact information as well as agree to our Terms of Services and Privacy Policy. The consent methodology used was based on the regulations of EU GDPR with all terms and conditions being available for the potential patients. Secondly, they were directed to a demographic questionnaire including disease etiology, and requested to upload photos of their perceived AK lesions.* The total spending for this online recruitment campaign via Meta was 14,285 USD i.e. 238 USD per randomized patient. The campaign was designed over the course of approximately 1.5 months by a team of developers, designers specializing in user experience (UX), and a recruitment team, and approved as part of COAKS by the Capital Region of Denmark's Ethics Committee.

### 2.2 Virtual eligibility review

The patient-obtained photos of potential AK lesions were assessed online by a team of 4 remote board-certified dermatologists with experience in teledermatology, and in combination with the data from the demographic questionnaire, possible study candidates were identified. The panel of dermatologists remained the same throughout the review process, from the virtual eligibility review until the end of the study. Images were shared using a GDPR-compliant software designed for use in teledermatology (DermView, Studies&Me, Copenhagen, Denmark). If an area of sun damage with at least one AK was visible on the submitted photographs, the participant qualified for an in-clinic examination. Patient-obtained photographs were assessed within 24 h. In case a significant skin disorder was detected, participants received an automated response to inform them that they may have a serious skin condition, are unable to participate in the study, and should seek urgent dermatological care.

### 2.3 In-clinic eligibility review

A virtual patient liaison team was responsible for contacting potential participants by phone and completing their bookings for the in-clinic visits for a physical exam at the trial site (Copenhagen University Hospital, DK). The patient liaison team consisted of 2 professionals with a background in nursing, mobile technology and user experience (UX) design, who were full-time employees during the recruitment phase of the study. Furthermore, patients received educational resources prior to their visit to prepare them for the study, including digital patient information leaflets and study-related video containing training material hosted on YouTube. At the in-clinic screening visit, the investigator obtained written informed consent before performing a physical exam and detailed medical history, and enrolled the patients if eligibility was confirmed for the COAK study (2021-000934-32; NCT05164393).

### 2.4 Analytics

The online tool Facebook Analytics (Meta, Dublin, IE) was used to track the online advertisement campaigns and generate the number of impressions, reach, and clicks for each campaign. These numbers were used to monitor the success of the different campaigns. A customized recruitment tool was used to track the number of times forms were filled out by users on the landing page (Studies&Me, Copenhagen, DK). Social media metrics evaluated in this study included the number of impressions, the reach, and the conversion rate (CR). Impressions were defined as the number of times an advertisement appeared on a users screen. Reach referred to the number of unique users who have visited one or several social media platforms included in our advertising campaign i.e. an individual with an anonymous IP address who has visited a social media platform connected to Meta regardless of the number of visits or platforms used. Lastly, the CR was defined as the percentage of potential participants, i.e. users who have undergone pre-eligibility evaluation, who were willing and eligible to be enrolled in the study.

**Fig. 2:**
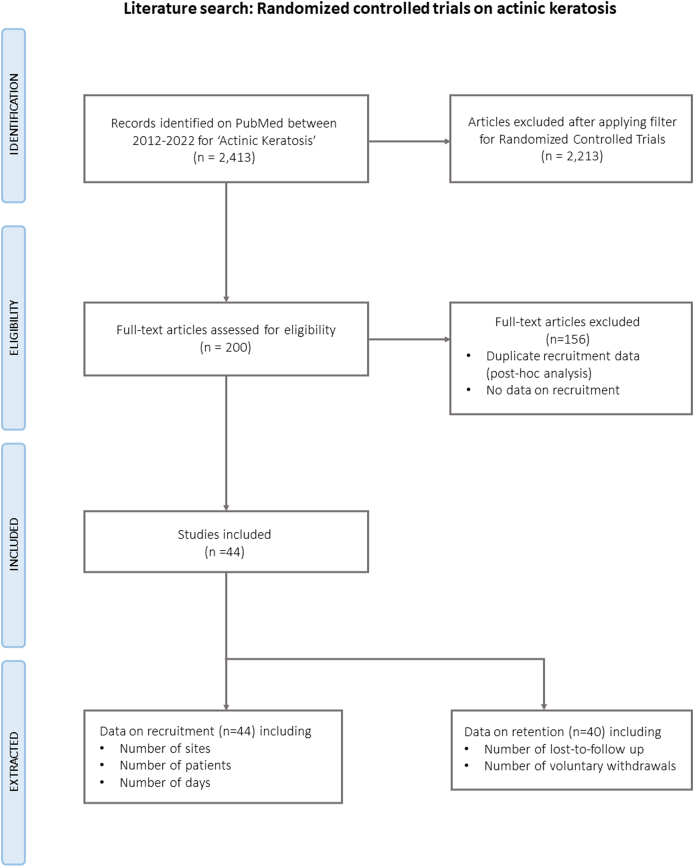
**Flow chart of the literature search for randomized controlled trials conducted in the past 10 years on actinic keratosis**. To calculate metrics on study performance, pre-filtered articles were reviewed (full text) to extract data on recruitment efficiency and retention rate.

*To evaluate the recruitment efficiency, the number of patients enrolled per day per site was reported and compared to available data extracted from recently published RCTs in patients with AK on PubMed using the following parameters: search terms ‘actinic keratosis’ or ‘actinic keratoses’, filter ‘RCT’, time frame 2012–2022. Only studies that reported on the duration of their recruitment period were included. Relative recruitment efficiency was calculated based on the average of the total productivity (number of included patients number of invested days) of the past 10 years divided by the productivity of the COAKS recruitment. Retention was defined as the number of patients who either completed a study or whose participation was terminated by the investigators i.e. excluding voluntary withdrawals by patients and lost to follow-up; retention ranged from 100 % (complete retention of all patients) to 0 % (no retention of any patients). The flowchart for the literature review can be found in*Fig. 2.

## 3 Results

In the period from the 28th of October to the 19th of December 2021, we completed a multi-step recruitment process for a single-center interventional RCT using a digital ecosystem. The target of enrolling 60 patients was achieved in 53 days using an online recruitment flow consisting of social media advertising and virtual eligibility review before in-clinic enrollment.

### 3.1 Social media metrics

**Fig. 3:**
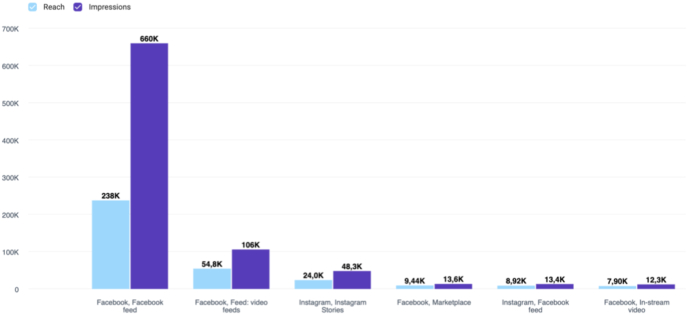
**Bar chart showing the breakdown of user and content views across Social Media platforms for patient recruitment.** Facebook feed (homepage news feed advertising) generated the highest reach (number of users) and number of impressions (content views), followed by Facebook video feed (stand-alone video advertising), Instagram stories (temporary image and video content with advertising), Facebook marketplace (digital marketplace advertising), Facebook feed on Instagram (cross-posted content with advertising), and Facebook in-stream video (video advertising delivered to users watching videos).

The number of times the campaigns were displayed on Facebook and Instagram (‘impressions’) was 886,670, and the total number of unique users exposed online to a campaign (the ‘reach number’) was 309,000. In total, potential study participants clicked the online advertisements 27,814 times. The impressions and reach for each advertising channel are shown in Fig. 3.

The advertisement that led to the highest number of enrolled patients (15 out of 60) and the lowest number of screen failures (30 %) addressed the opportunity to be seen by a dermatologist, access to new treatments for AK, and affiliation with Copenhagen University Hospital. In comparison, the Facebook advertisement that led to the highest proportion of screen failures (66.7 %) had an altruistic message of contributing to science.

### 3.2 Virtual eligibility

A total of 837 users signed up for the study by providing their contact information, corresponding to 3 % of all advertisement clicks. Of the 556 subjects (66.4 %) who filled in the demographic questionnaire, 401 (72.0 %) were willing to share pictures of their self-diagnosed AK-lesion for further eligibility review, and of these, 140 (25.0 %) were subsequently considered by the online dermatologists to be eligible for the COAK study. The patient liaison team requested 80 (20.0 %) of the 401 potential candidates to re-take photographs due to low image quality.

Supplementary material 3 shows the main reasons for excluding patients based on the answers provided in the questionnaire. The most common exclusion criteriion of the 556 subjects was lack of an AK diagnosis (29 %), which was further reviewed by remote dermatologists based on the provided photographs, followed by smartphones incapable of supporting the web-based study application for COAKS (n = 49; 8.8 %). The most common reasons for rejection based on photographs (n = 351) were lack of visible AK (21.3 %), other visible skin diseases (15.2 %), low image quality after re-take (6 %), or AK lesion outside of the target area (5.1 %). The average number of potential participants per week was 17.5 users per week.

### 3.3 In-clinic eligibility and enrollment

Given the total of 556 users (395 women, 161 men) who showed enough interest in participating in the trial to provide their demographic information, our recruitment campaigns achieved a conversion rate (CR) of 10.8 % (60/556). A closer evaluation of the age and gender distribution of potential participants showed that higher age and male gender positively influenced the CR. Users in the age bracket of 65–80 years were more likely to show sustained intersted in participating in the trial and to fulfill all inclusion criteria, resulting in a conversion rate of 16.8 %.

Of the 140 potential participants considered eligible, 115 (82 %) were scheduled for site visits; 18 % chose to withdraw during the booking phone call. Ninety out of 115 scheduled visits at the site (78%) were conducted before completing the recruitment goal (Fig. 1). The in-clinic enrollment success rate was 66.6 % i.e. 90 site visits were conducted to reach 60 fully randomized patients (60/90), resulting in a screen failure of 33.3 % due to either no (10 %) or insufficient (73.3 %) AK lesions, or undergoing treatment for AK (16.7 %). Table 1 shows the demographic trends of the eligible and randomized participants and the effect of age and gender on the CR. The likelihood of a patient showing sustained interest in participating in the study increased with the age of patients, with the highest response rate in the age bracket of 65–80 years (CR 16.8 %). Further, the CR was approximately 3 times higher for male (CR 20.4%; 33/161) than for female users (CR 6.8%; 27/395).

**Table 1:** Demographic overview of interested users, potentially eligible volunteers and clinical trial patients.

	Total	Female	Male	18–24	25–34	35–44	45–54	55–64	65–80	80+
Sign ups	837	n.a.	n.a.	1 (0.1 %)	8 (1 %)	38 (4.5 %)	165 (19.7 %)	257 (30.7 %)	368 (44 %)	0 (0 %)
Eligibility-reviewed	556	395 (71 %)	161 (29 %)	1 (0.2 %)	3 (0.5 %)	21 (3.8 %)	111 (20 %)	170 (30.6 %)	250 (45 %)	0 (0 %)
Randomized	60	27 (45 %)	33 (55 %)	0 (0 %)	0 (0 %)	0 (0 %)	3 (5 %)	15 (25 %)	42 (70 %)	0 (0 %)
Conversion rate (%): (eligible to randomized)	10.8 %	6.8 %	20.4 %	0 %	0 %	0 %	2.7 %	8.82 %	16.8 %	0 %

### 3.4 Recruitment efficiency and retention

Based on 44 studies included in our PubMed search (Fig. 2), the average RCT on AK has 4.6 sites (SD 7.2) which included 116 patients (SD 167) who were recruited within 401 days (SD 284). By enrolling 60 patients over the course of 53 days, we spent approximately 0.9 days to include one patient i.e. a yield of 1.13 patients per day per site. When comparing the recruitment efficiency of our study to the existing literature, the results indicate a higher efficiency both in total and across sites of 281.0 % and 928.9 %, respectively i.e. 9 times more efficient. The COAKS trial reported a lost-to-follow up rate of 10 % (54/60 patients) i.e. a retention rate of 90 %. When excluding patients who withdrew due to side effects (n = 1) or were terminated early due to protocol breach (n = 1), our adjusted retention rate corresponded to the average retention rate of 95 % (SD 8 %), which was reported in 40 out of 44 studies The recruitment and retention metrics are summarized in a heatmap in Table 2.

**Table 2:**
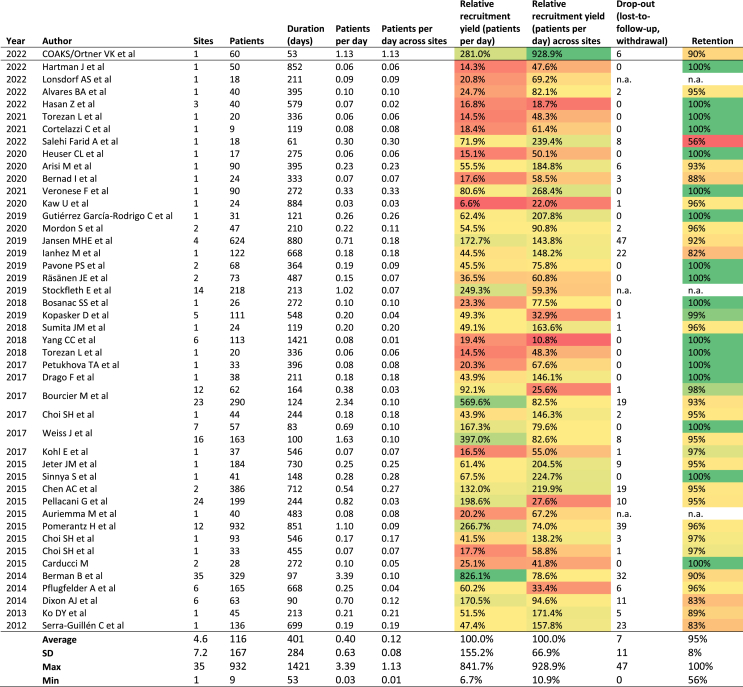
**Overview and performance heatmap of recruitment metrics extracted from 44 randomized clinical trials on actinc keratosis published 2012**–**2022.** The online recruitment approach using social media was the most efficient in relation to the average number of patients per day across sites and achieved a retention rate of 90 %.

Data extracted from pubmed applying the RCT filter using the search term ‘actinic keratosis’.

^∗∗^Defined as voluntary withdrawals and lost to follow-up to measure retention color-coded from lowest (white) to highest (green) values.

## 4 Discussion

By employing a digital ecosystem consisting of social media for targeted advertising and patient-obtained smartphone photographs for remote assessment, our online recruitment approach for a GCP-monitored randomized controlled Phase I/IIa clinical trial reached the initial recruitment target of 60 patients with AK in under 8 weeks. With a daily enrollment of 1.13 patients per day, this first report on online recruitment of AK was more than 9 times as efficient as RCTs from the past 10 years. Our retention rate of 90 % closely matches the average literature-reported patient retention of similar offline-recruited trial populations and corresponds to previous reports on higher-than-expected adherence to teledermatological services [4].

Efficient recruitment remains a key factor in allocating trials to clinical research organizations [5]. In a study by Dombernowsky et al., 84 % of industry representatives from Nordic countries agreed that efficient recruitment is the most valued site-related quality. Their survey further showed that the pharmaceutical industry views trial expenses as secondary to timely recruitment of trial patients [6]. To accelerate conventional recruitment and reach recruitment targets, investigators often use static advertising tools such as flyers as well as electronic health records (EHR), including patient registries and databases [7]. In a recent study on industry-sponsored clinical trials, EHR were used in 85 % of surveyed Nordic trials, which is far above the 11 % reported globally [8]. Recruitment goals in Northern Europe, however, were only reached in half of the included studies. Despite initial concerns that online recruitment of an AK patient population, consisting of primarily older individuals, could be challenged by a lack of interest in social media and the COVID-19 pandemic and related restrictions, our study succeeded without the use of EHR, conventional broad marketing, or any form of in-clinic patient referral. When comparing online recruitment to traditional offline recruitment strategies, online strategies appear more time-efficient. Our review of the published literature on AK from the past 10 years highlighted the time efficiency of an online recruitment approach for RCTs in dermato-oncology (Table 2) [[9], [10], [11], [12], [13], [14], [15], [16], [17], [18], [19], [20], [21], [22], [23], [24], [25], [26], [27], [28], [29], [30], [31], [32], [33], [34], [35], [36], [37], [38], [39], [40]]. A review by Jacques RM et al. of the recruitment rates using conventional strategies in 388 RCTs between 1997 and 2020 found that the median patient recruitment rate per site was 0.95 per month (0.03 per day) [41]. In our study, 60 patients were enrolled at a single site over a period of 52 days (1.13 per day), corresponding to 34.6 patients per month, thereby being more than 36 times more efficient than the average recruitment rate for RCTs. The driving force behind the high recruitment speed using online strategies is the sheer number of social media users exposed to trial advertisements. In this study, a total of 309,000 users were exposed to advertisements that generated 27,814 clicks i.e. an average daily engagement of 534 potential participants. This superiority in capacity comes with the trade-off of user segments who wrongly identify as potentially eligible, creating a theoretical screening burden that can be buffered by targeted advertising using relevant filters on social media platforms. While decentralization comes with an overall increase of cost of training and educating both staff and participants as well as investments in social media marketing and data sharing platforms compliant with ICH GCP guidelines [42], total savings compared to conventional studies can be significant during study execution. As the patient information workflow is lean-optimized, remote dermatologists were able to evaluate up to 30 patients per hour cutting clinical evaluation time to approximately 10 %.

Despite the clear advantages of targeted advertising using social media, their perceived impact on users' privacy and incompatibility with the European Union's General Data Protection Regulation (GDPR) has led to legal consequences for tech giants such as Meta [43]. In practice, filtering social media users based on their interests in specific diseases or treatments to make advertising user-targeted requires the use of ‘data concerning health’ and, consequently, explicit informed consent to have sensitive health data processed. The GDPR framework necessitates providers to operate on an opt-in basis, which can limit the pool of users that choose exposure to targeted advertising and, thereby online recruitment. National guidelines published by the Danish Medicine's Agency endorse the use of digital solutions for decentralized trial activities and provide a regulatory framework that allows for GDPR-compliant recruitment strategies. While debates on the most suitable lawful basis to consent to data processing are ongoing, our herein-described recruitment flow and advertising practices may necessitate devising broader search strategies if they were to be adopted in the future, such as using indirect markers of AK e.g. interest in skin care, sun protection, or beach vacations. A larger recruitment funnel would inevitably increase the volume of potentially interested users, underscoring the importance of a pre-eligibility check, including online questionnaires addressing in- and exclusion criteria and remote assessment of patient-obtained smartphone photographs.

Using a combination of targeted social media advertising and patient-obtained smartphone photographs to recruit for a GCP-monitored RCT investigating low-grade AK has not previously been reported. While AK count and grading may be of higher importance in a research than clinical setting, the use of teledermatology has been studied insufficiently to draw conclusions on its reliability [44]. In our study, the large majority of social media users attached a photo of their suspected AK lesions after filling in the screening questionnaire. Nonetheless, it remained a challenge to make a correct evaluation of the precise number of AK, as a lack of lesions was the reason for 73.3 % of screen failures. Our experience and previous studies on remote assessment suggest that, in contrast other dermatological conditions, in-clinic lesion inspection and palpation may be pivotal for the diagnosis and grading of AK [45,46]. Consequently, continuous feedback to remote assessors and re-education on the remote digital assessment criteria were crucial to mitigate the potentially incorrect AK diagnosis at the pre-eligibility check stage.

Our work has some limitations. Given the target disease and its age prevalence, a more appropriate age cut-off may have helped reduce the burden on the pre-evaluation team and total spending without decelerating recruitment. Further, removing the language filter may have increased the number of potential participants with AK since many expats and Scandinavian nationals may use non-Danish default settings despite fluency in the language. Further, online recruitment may reach even higher efficiency when coupled with either increased in-clinic capacity, allowing for extended opening hours for in-clinic enrollment, or a fully digital workflow relying solely on virtual assessments. Finally, inaccurate self-assessment of potential AK lesions could have been avoided with a patient education segment to clarify the differences between AK and other sundamage-related skin disorders, including dyspigmentaiton and seborrheic keratosis. Lastly, no clear conclusions can be drawn on the performance of specific advertisements. Future studies should include the use of a patient focus group to explore themes and value propositions that can incentivize participation in RCTs and permit evidence-based targeted advertising.

In conclusion, combining targeted social media advertising with remote pre-eligibility assessments using patient-obtained smartphone photographs proved to be an effective way to recruit patients online for an interventional RCT on a new topical treatment for AK. Our study indicates that the use of digital solutions may be a suitable alternative to conventional recruitment methods in research settings where time is of the essence. Further investigations of online recruitment are needed to confirm the potential scalability of our approach and its translatability to areas outside of dermatology.

## Funding sources

This study was funded by Coegin Pharma AB and Copenhagen University Hospital, Bispebjerg.

## Author contributions statement

Study conception and design: Budnik, Manole, Zibert, Haedersdal, Acquisition of data: Ortner, Budnik, Manole, Laugesen, Havsager, Analysis and interpretation of data: Ortner, Budnik, Manole, Laugesen, Haedersdal.

Drafting of manuscript: Ortner, Laugesen.

Critical revision: Ortner, Budnik, Manole, Zibert, Laugesen, Havsager, Haedersdal.

## Declaration of competing interest

The authors declare that they have no known competing financial interests or personal relationships that could have appeared to influence the work reported in this paper.

JRZ is a paid consultant to Coegin Pharma AB; OB, SH, and CAPL are employees at S&Me; IM is a paid consultant to S&Me; MH has received a research grant from S&Me.

## Data Availability

Data will be made available on request.
