# Supplementary material for: Accelerating patient recruitment using social media: Early adopter experience from a good clinical practice-monitored randomized controlled phase I/IIa clinical trial on actinic keratosis
Source: Contemp Clin Trials Commun. 2023 Dec 20;37:101245. doi: 10.1016/j.conctc.2023.101245 (PMC10792556; doi:10.1016/j.conctc.2023.101245)
Supplement: Multimedia component 1 [file mmc1.docx]

**Supplementary material 1: CONSORT flow chart of the Copenhagen Actinic Keratosis study (COAKS).**

**
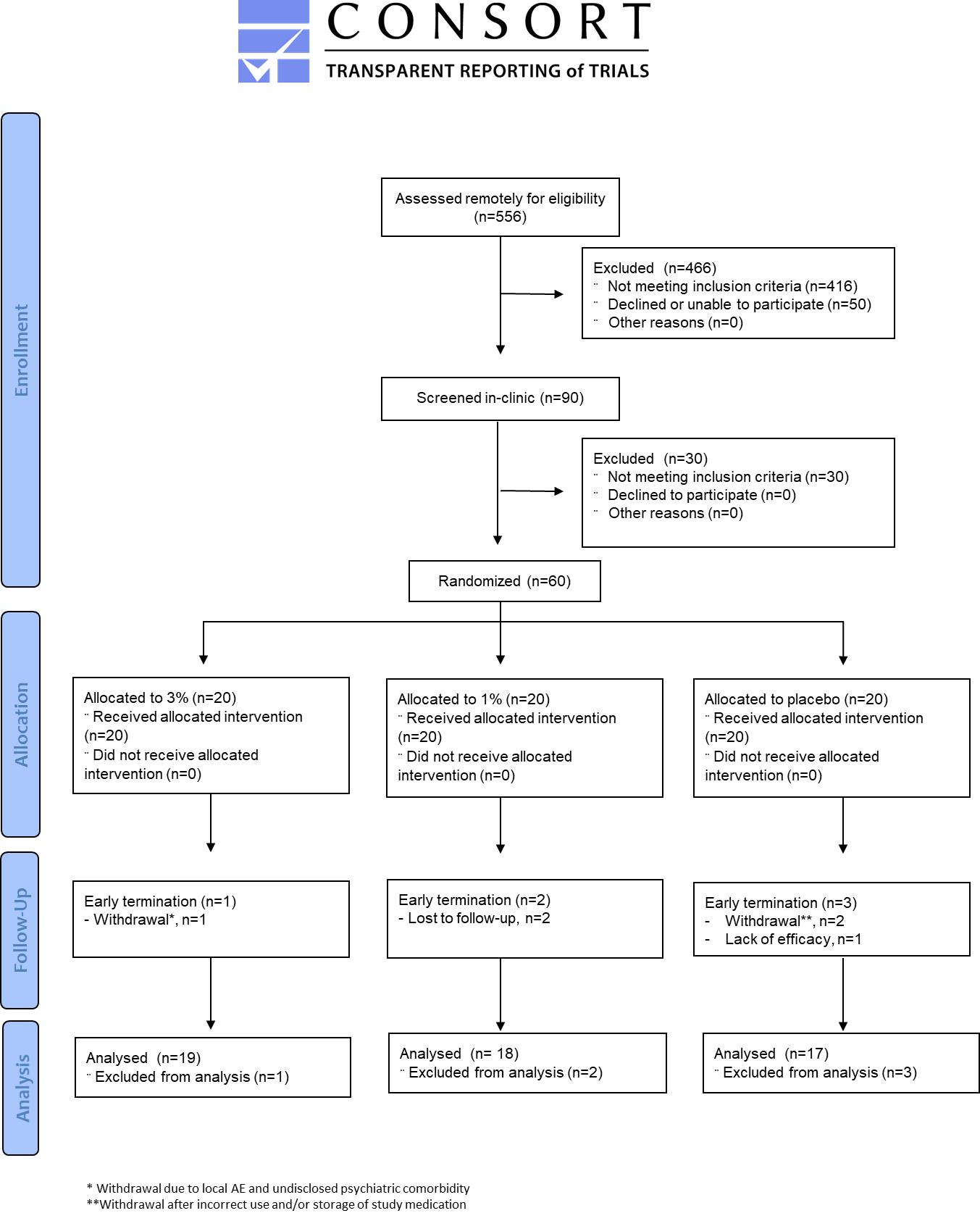
**

**Supplementary material 2:** Overview of filter settings applied in an online social media advertising campaign.

| Filter categories | Setting | Rationale |
| --- | --- | --- |
| Gender | Male and Female | To avoid gender bias (no exclusion criteria) |
| Age | >18 (18-65+ filter) | To avoid age bias (no exclusion criteria) |
| Target area | 75 km distance to Copenhagen University Hospital - Bispebjerg (excluding Sweden) | To minimize transportation time for patients and reduce study spending on reimbursements |
| Language | Danish | All study material and documentation was in Danish |
| Device (interests) | Iphone 7, Nexus 5 | Sufficiently modern smartphone to perform recruitment and study tasks |
| Device (behaviors) | Facebook access (mobile): Android devices, Owns: iPhone 7, Owns: iPhone 7 Plus, Facebook access (mobile): iPhone 8, Facebook access (mobile): iPhone 8 Plus, Facebook access (mobile): iPhone X, Facebook access (mobile): iPhone XS, Facebook access (mobile): iPhone XS Max or Facebook access (mobile): iPhone XR |  |
| Interests | Sunburn, Skin Cancer/Melanoma Awareness, Sunscreen, Cryotherapy, Dermatology, Skin Cancer Foundation, Skin, Exfoliation (cosmetology), Skin care | To find suitable patients |

**Supplementary material 3.** Reasons for excluding potential study participants based on answers to the pre-screening questionnaire.

| **Pre-filtering questions** | **Yes** | **No** |
| --- | --- | --- |
| Diagnosis of AK | 70.3% (n=391) | 29.7% (n=165) |
| Smartphone that supports the webapp | 91.2% (n=507) | 8.8% (n=49) |
| Approved method of contraception | 94.6% (n=526) | 5.4% (n=30) |
| No immunosuppressive treatment | 94.6% (n=526) | 5.4% (n=30) |
| No allergy to sea food | 98.4% (n=547) | 1.6% (n=9) |
| No organ transplant recipient | 99.6% (n=554) | 0.4% (n=2) |
| Danish language fluency | 99.8% (n=555) | 0.2% (n=1) |
